# Accuracy of multi-trait genomic selection using different methods

**DOI:** 10.1186/1297-9686-43-26

**Published:** 2011-07-05

**Authors:** Mario PL Calus, Roel F Veerkamp

**Affiliations:** 1 Animal Breeding and Genomics Centre, Wageningen UR Livestock Research, 8200 AB Lelystad, The Netherlands

## Abstract

**Background:**

Genomic selection has become a very important tool in animal genetics and is rapidly emerging in plant genetics. It holds the promise to be particularly beneficial to select for traits that are difficult or expensive to measure, such as traits that are measured in one environment and selected for in another environment. The objective of this paper was to develop three models that would permit multi-trait genomic selection by combining scarcely recorded traits with genetically correlated indicator traits, and to compare their performance to single-trait models, using simulated datasets.

**Methods:**

Three (SNP) Single Nucleotide Polymorphism based models were used. Model G and BCπ0 assumed that contributed (co)variances of all SNP are equal. Model BSSVS sampled SNP effects from a distribution with large (or small) effects to model SNP that are (or not) associated with a quantitative trait locus. For reasons of comparison, model A including pedigree but not SNP information was fitted as well.

**Results:**

In terms of accuracies for animals without phenotypes, the models generally ranked as follows: BSSVS > BCπ0 > G > > A. Using multi-trait SNP-based models, the accuracy for juvenile animals without any phenotypes increased up to 0.10. For animals with phenotypes on an indicator trait only, accuracy increased up to 0.03 and 0.14, for genetic correlations with the evaluated trait of 0.25 and 0.75, respectively.

**Conclusions:**

When the indicator trait had a genetic correlation lower than 0.5 with the trait of interest in our simulated data, the accuracy was higher if genotypes rather than phenotypes were obtained for the indicator trait. However, when genetic correlations were higher than 0.5, using an indicator trait led to higher accuracies for selection candidates. For different combinations of traits, the level of genetic correlation below which genotyping selection candidates is more effective than obtaining phenotypes for an indicator trait, needs to be derived considering at least the heritabilities and the numbers of animals recorded for the traits involved.

## Background

Due to the availability of affordable genome-wide dense marker maps, the use of marker information in practical animal and plant breeding programs is increasing. In particular, the application of genomic selection is becoming the new standard in animal breeding e.g. [1,2], and is an emerging alternative for marker-assisted selection in plant breeding [3,4]. Genomic selection uses genome-wide dense marker maps to accurately predict the genetic ability of an animal, without the need of recording phenotypic performance of its own or from close relatives, such as sibs or offspring e.g. [5]. Genome-wide prediction is also being recognized as an important tool to predict phenotypes [6] and genetic risk for diseases [7] in other fields than animal or plant breeding. The key principle for all these applications is the simultaneous estimation of all genome-wide marker effects based on a reference population with known phenotypes. Many different models have been proposed to simultaneously estimate marker effects [2,8]. Most of the proposed models try to reduce the effective dimensionality of the marker data, since the number of markers is typically much larger than the number of phenotyped animals in the reference population. Reduction of dimensionality of the markers, i.e. whether a locus affects the trait or not, is often integrated in the sampling process using model selection [9,10]. An added benefit of such integrated marker selection procedures is that posterior distributions are provided for the probability that a locus affects a trait, and these can be used for QTL (Quantitative Trait Loci) mapping purposes [11].

By putting emphasis on loci that are closely linked to causative loci, genomic prediction holds the promise to be particularly beneficial for selection on traits that are difficult or expensive to measure, that are sex-linked, or that are expressed late in life. One effective strategy that has been used to deal with such traits in the past, without using genotypic information, has been the implementation of multi-trait prediction with indicator traits that are easier or cheaper to record. These might be closely linked traits, for example somatic cell count as indicator trait of mastitis, or the same trait recorded in a different environment or country. Multi-trait prediction allows to use information simultaneously from relatives and from different traits [12]. Therefore, an important question is to evaluate what is the added value of including genomic information in multi-trait genomic prediction.

The objectives of this paper were to develop methods for multi-trait genomic breeding value prediction, to enable multi-trait genomic selection, and to compare the accuracy of prediction among the different methods and with equivalent single-trait models, based on the results of applications to simulated datasets.

## Methods

### Simulation

Datasets were simulated to compare the different models, in terms of accuracy of predicted breeding values. An effective population size of 500 animals was simulated, including 250 females and 250 males. This structure was kept constant for 1000 generations. Mating was performed by drawing the parents of an animal randomly from the animals of the previous generation. In total, 25 replicated datasets were simulated.

The simulated genome spanned 5 M (Morgan). Ten thousand bi-allelic loci were simulated across five chromosomes, with equal 0.05 cM distances between adjacent loci. In the first generation, animals received at random alleles 1 or 2 with equal chance. In the 1000 generations thereafter, each locus had a mutation rate of 2.5 × 10^-5^, so that a mutation drift balance was reached within a limited number of generations [13]. A mutation caused allele 1 to become allele 2, and vice versa. Genotypes from the last four generations, as well as pedigree information of the last six generations, were retained for analysis. In total, on average across replicates, 5,655 loci segregated in the last four generations. These four generations will hereafter be referred to as generations 1 to 4.

Two hundred loci segregating in generations 1 to 4 and evenly distributed across the genome, were drawn to be QTL loci. These QTL were used to simulate two traits, with heritabilities of 0.9 and 0.6, reflecting average offspring performances such as daughter yield deviations [14] or de-regressed proofs [15]. For example, if one considers that the animals in the reference population reflect dairy bulls each with 100 daughters and their phenotypic records, the chosen heritabilities of 0.6 and 0.9 correspond to traits with heritabilities at the phenotypic level of 0.06 and 0.33, respectively, i.e. a fertility and a production trait in dairy cattle. The heritabilities of 0.6 and 0.9 were derived using the formula  e.g. [16], where  is the reliability of selection (in this case the heritability used to simulate the phenotypes of the animals in the reference population), *n *is the number of daughters and *h^2 ^*is the heritability at the phenotypic level. The two traits were simulated by drawing the allele substitution effects of each QTL locus from a multivariate normal distribution that followed the simulated genetic correlation. Three genetic correlations were considered, i.e. 0.2, 0.5, or 0.8.

### Scenarios

To investigate the ability of the models to predict breeding values for animals with records for the two traits, only one, or none of the traits, two scenarios were considered differing in the number of animals that had phenotypes available for each of the traits (Table 1). In scenario 1, all animals in generations 1 and 2 had phenotypes for both traits. In scenario 2, all animals in generation 1 had phenotypes for both traits, while one half of the animals of generation 2 had phenotypes for the first, and the other half of the animals had phenotypes for the second trait. In both scenarios, all the animals in generations 3 and 4 had no phenotypes for either trait, and thereby reflected juvenile selection candidates.

**Table 1:** Numbers of animals with phenotypes per generation and scenario

Scenario	Generation	Trait 1	Trait 2
1	1	500	500
	2	500	500
	3	0	0
	4	0	0

2	1	500	500
	2	250^1^	250^1^
	3	0	0
	4	0	0

^1^In scenario 2, half of the animals in generation 2 have a phenotype for trait 1, while the other half have a phenotype for trait 2

### Models

Four different models were used to estimate breeding values. The general multi-trait model was:

where *y_ij _*is the phenotypic record for trait j of animal *i, μ_j _*is the overall mean for trait *j, animal_ij _*is the random polygenic effect of animal *i *for trait *j, SNP_ijkl _*is a random effect for allele *l *on trait *j *at locus *k *of animal *i*, and *e_ij _*is a random residual for animal *i*.

The first model omitted the SNP effects, and used a relationship matrix based on the pedigree retained to estimate the polygenic effects and the polygenic (co)variances of traits 1 and 2 (model A). The second model was the same as the first model, but included a genomic relationship (**G**) matrix calculated by using all the markers to estimate the polygenic effects (model G). This **G **matrix was calculated as described by VanRaden [17]:

where *p_i _*is the frequency of the second allele at locus *i*, and **Z **is derived from genotypes of all included animals, by subtracting 2 times the allele frequency expressed as a difference of 0.5, i.e. 2(*p_i _*- 0.5), from matrix **M **that specifies the marker genotypes for each individual as -1, 0 or 1. Here, we used allele frequencies of 0.5 that reflected allele frequencies in the base generation i.e. in the very first generation of the simulation.

The third and fourth models included both a polygenic effect with a pedigree-based relationship matrix, and SNP effects. The difference between the third and fourth model resulted from considering one (model 3) or two (model 4) distribution(s) for the SNP effects.

SNP effects, in the general model denoted as *SNP_ijkl_*, were estimated in models 3 and 4 as *q_ijkl _× v_jk _*, according to Meuwissen and Goddard [11], where *q_ijkl _*is the size of the effect of allele *l *at locus *k *and *v_jk _*is a scaling factor in the direction vector for locus *k *that scales the effect at locus *k *for trait *j*. In the original implementation by Meuwissen and Goddard [11], the variance of the direction vector *v_.k_*, denoted as **V**, is sampled per locus for each trait *j *separately, without considering covariances between the traits across loci. Here, in both models 3 and 4 and for the estimation of **V**, covariances between traits across loci are considered. Therefore, the prior distribution for **V **in this case was, according to Meuwissen and Goddard [11]:

where **S**_**0**(*no*) _was chosen such that it reflected the total genetic (co)variance between traits *n *and *o*, divided by the total number of SNP. **V.. **was sampled from the following conditional *m *variate-inverted Wishart distribution with (*nloc *+ 10) degrees of freedom:

where , *nloc *= number of evaluated marker loci, and 10 is the number of degrees of freedom for the prior distribution.

Model 4 was similar to model 3, but included a QTL-indicator (*I_k_*) for each bracket, that had a value of either 0 or 1. According to Meuwissen and Goddard (2004), in this case the prior distribution of **V.. **is similar to that from model 3, but here **S_0(..) _**was chosen such that it reflected the total genetic (co)variances of traits *n *and *o*, divided by the total number of expected QTL instead of the number of SNP. Furthermore, **V.. **was sampled from an inverted Wishart distribution as described above for model 3, but in this case:

Where the QTL-indicator *I_k _*was sampled from:

where *p_k _*is the prior QTL probability, i.e. the probability that *I_k _*is equal to 1, which follows a Bernoulli distribution. Prior QTL probabilities used in the analyses reflected the prior assumption that 100 QTL underlie both traits.

The third model is referred to as model BCπ0, since this model is similar to a model that is termed BayesCπ0 [18]. The fourth model is referred to as Bayesian Stochastic Search Variable Selection (BSSVS) e.g. [10].

In all the models, the residuals were assumed to be normally distributed *N*(**0**, **R**), where **R **is the *m *× *m *residual covariance matrix. In models A, BCπ0 and BSSVS, the polygenic values were assumed to be normally distributed *N*(**0**, **A **⊗ **G_A_**), where A is the additive relationship matrix and **G_A _**is the *m *× *m *polygenic covariance matrix. Matrices **R **and **G_A _**were both sampled in the Gibbs sampler from an inverted Wishart distribution, with a uniform prior distribution.

Models A, BCπ0 and BSSVS were performed using Gibbs sampling with residual updating. Model A was run for 5,000 cycles, discarding 2,000 cycles for burn-in. Models BCπ0 and BSSVS were run for 10,000 cycles, discarding 2,000 cycles for burn-in. Except for the multi-trait runs in the second scenario where 30,000 cycles were run with 10,000 cycles discarded for burn-in, since initial results showed that more cycles were required for convergence in that scenario. Model G was performed using ASReml [19], because initial analyses using the Gibbs sampler showed slow convergence of the genetic variances for scenario 2.

In the multi-trait analyses of scenario 2 for the models that were analyzed using the Gibbs sampler, residuals for missing phenotypes in generation 2 were sampled using an EM algorithm. The missing residuals were drawn from the following distribution, according to VanTassell and VanVleck [20]:

where *m *stands for missing and *o *for observed records. This allowed us to sample the effects in the model using residual updating. Residual (co-)variance matrices were estimated conditional only on residuals linked to observed records.

Each simulated dataset and scenario were analyzed three times with all four models: first traits 1 and 2 were analyzed separately in a single-trait (ST) model, and then both traits were analyzed together in a multi-trait (MT) model.

### Comparison of methods

The results of each of the different models were evaluated using the accuracy of predictions and the bias of the estimates. Accuracy of prediction was calculated as the correlation between simulated and estimated breeding values. Using t-tests, the significances of differences were investigated between the accuracy obtained with different SNP-based models both within ST and MT models, and between the same SNP-based models in ST and MT application. Bias was assessed by regression of the simulated on estimated breeding values. In addition, (co)variances of the estimated breeding values were compared to those of the simulated breeding values, to assess the ability of th models to capture the true genetic (co)variances.

## Results

In generations 1 to 4 of the simulated data, the linkage disequilibrium between adjacent markers, measured as *r^2 ^*[21], was 0.32. The realized correlations between the simulated breeding values of the two traits were on average 0.25, 0.54 and 0.75. Hereafter, we will refer to those correlations as being the simulated genetic correlations.

### Single-trait models

**Figure 1:**
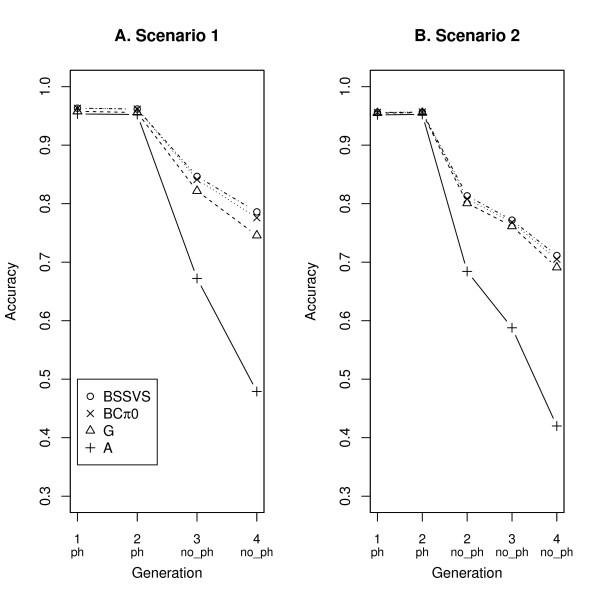
**Accuracies for trait 1 from all four single-trait models**. Displayed accuracies are for both scenarios across generations with animals with (ph) and without phenotypes (no_ph).

**Figure 2:**
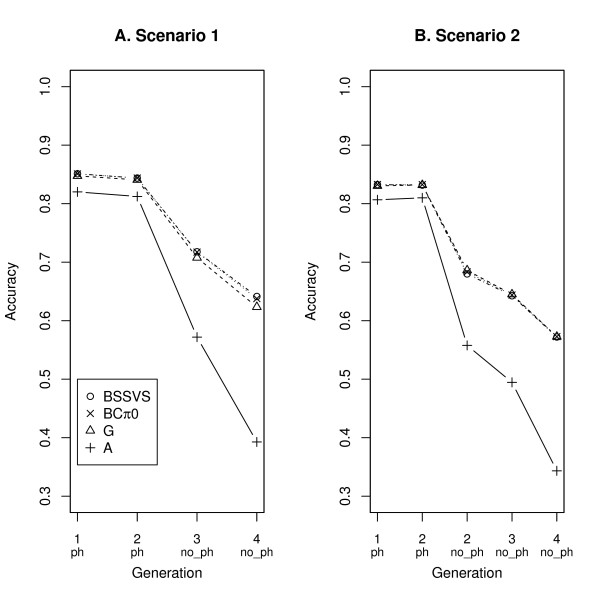
**Accuracies for trait 2 from all four single-trait models**. Displayed accuracies are for both scenarios across generations with animals with (ph) and without phenotypes (no_ph).

In Figures 1 and 2, the accuracies are given for all ST models, per trait and per scenario. For the first trait, the accuracy of model BSSVS was larger than that of model BCπ0 that was in turn larger than that of model G and all were considerably larger than the accuracy of model A (Figure 1A). When omitting the 250 phenotypes from generation 2 (scenario 2), all accuracies for trait 1 decreased, and the differences between SNP-based models disappeared (Figure 1B). For the second trait, in scenario 1 the order of accuracies was similar to that for trait 1, but differences were smaller (Figure 2A). In the case of scenario 2, the accuracy decreased for all animals, but especially for those without phenotypes (Figure 2B). In all scenarios, the ST models including SNP information yielded similar accuracies, and showed a comparable decrease in accuracy when the distance to the phenotyped animals became larger (i.e. from generation 3 to 4). Only for trait 1 in scenario 1, based on the standard errors of the estimates across replicates, were the accuracies of the different SNP-based models for juvenile animals in generation 3 significantly different from each other (Table 2).

**Table 2:** Significance of differences in accuracies between all SNP models

Model	Scenario	**r**_**g**_^**1**^	Trait	G vs. BCπ0	G vs. BSSVS	BCπ0 vs. BSSVS
ST	1		1	***	***	
	1	0.25	2			
	1	0.54	2			
	1	0.75	2			
	
	2		1			
	2	0.25	2			
	2	0.54	2			
	2	0.75	2			

MT	1	0.25	1	***	***	***
	1	0.54	1	***	***	***
	1	0.75	1	***	***	***
	1	0.25	2	***	***	*
	1	0.54	2	***	***	*
	1	0.75	2	***	***	*
	
	2	0.25	1	***	***	
	2	0.54	1	***	***	**
	2	0.75	1	***	***	**
	2	0.25	2	***	***	
	2	0.54	2	***	***	
	2	0.75	2	***	***	

Comparisons are between ST and MT models for animals without any phenotypes (generation 3) between pairwise SNP-based models across scenarios, genetic correlations (r_g_), and traits

^1^Phenotypes for trait 1 were the same across genetic correlations, and therefore analyzed only once with each ST model; *** P-value < 0.001, ** P-value < 0.01, * P-value < 0.05

### Multi-trait models

**Figure 3:**
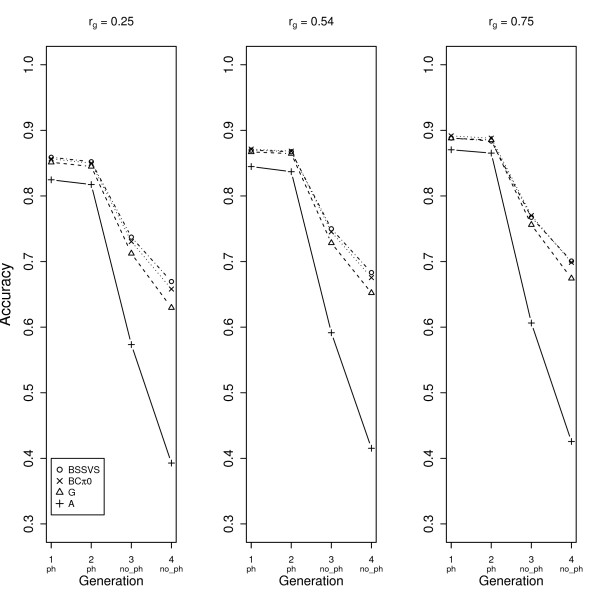
**Accuracies for trait 2 for scenario 1 for all four multi-trait models**. Displayed accuracies are across generations with animals with (ph) and without phenotypes (no_ph), with genetic correlations between both traits of 0.25 (A), 0.54 (B) and 0.75 (C), respectively.

The accuracies of all MT models for trait 1, in both scenarios, were similar to those of ST models. In Figure 3, the accuracies are shown for trait 2 for scenario 1, considering different genetic correlations with trait 1. The order of accuracies was similar across different genetic correlations (BSSVS > BCπ0 > G > > A), and differences between models were in all cases significant (Table 2). For animals without phenotypes, the accuracy increased from 0.03 to 0.04 across models when the genetic correlation increased from 0.25 to 0.75 (Figures 3A, B and 3C).

**Figure 4:**
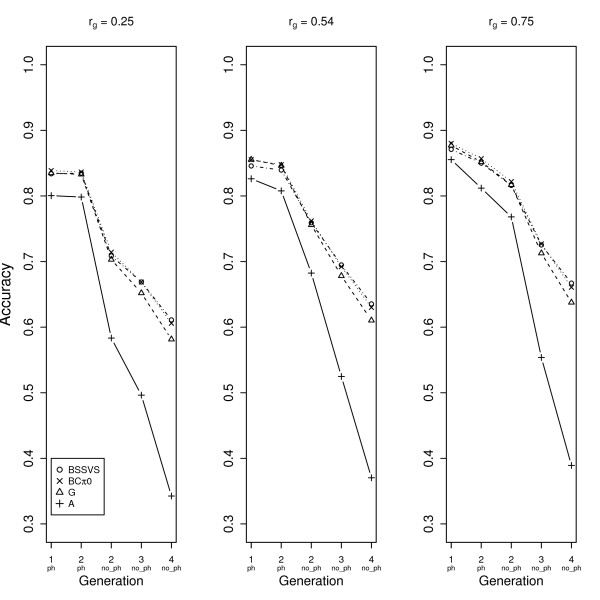
**Accuracies for trait 2 for scenario 2 for all four multi-trait models**. Displayed accuracies are across generations with animals with (ph) and without phenotypes (no_ph), with genetic correlations between both traits of 0.25 (A), 0.54 (B) and 0.75 (C), respectively.

In Figure 4, the accuracies are given for trait 2 and scenario 2, considering different genetic correlations with trait 1. In this case, for animals without phenotypes the order in terms of accuracies was BSSVS > BCπ0 > G > > A for all genetic correlations. The differences between BCπ0 and BSSVS were small and not significant (Table 2). Differences between G and BCπ0, and G and BSSVS were always significant (Table 2). Accuracies for trait 2 increased from 0.07 to 0.14 for the SNP-based models when the genetic correlation increased from 0.25 to 0.75. For animals with phenotypes, accuracies of the SNP-based models were very similar.

### Single- versus multi-trait models

Tables 3 and 4 show the increase in accuracy when changing from ST to MT models in scenarios 1 and 2, respectively for traits 1 and 2. In scenario 1, MT models did not increase accuracies for trait 1 compared to ST models (Table 3). In scenario 2, the accuracies for trait 1 were not increased by the MT models for animals with phenotypes. For animals without any phenotypes, the accuracy increased to a maximum of 0.01 for model A and 0.03 for the SNP-based models. For animals with phenotypes for trait 2, the accuracy increased to a maximum of 0.04 both for model A and the SNP-based models. Only in a few situations with a genetic correlation of 0.25, did the MT models yield slightly lower accuracies for trait 1 compared to the ST models. Accuracies of SNP-based models for trait 1 obtained with the MT models were only significantly higher than those from the ST models in scenario 2 when the genetic correlation was 0.75 (Table 5).

**Table 3:** Increase in accuracy comparing MT to ST models for trait 1

		Scenario 1				Scenario 2				
		
		**1**^**1**^	2	3	4	1	2	2	3	4
Model	**r**_**g**_	**1&2**^**2**^	1&2	no	no	1&2	1	no	no	no
	0.25	0.00	0.00	0.00	0.00	0.00	0.00	-0.02	-0.01	-0.01
A	0.54	0.00	0.00	0.00	0.00	0.00	0.00	0.01	0.00	0.00
	0.75	0.00	0.00	0.00	0.00	0.00	0.00	0.04	0.01	0.01

	0.25	0.00	0.00	0.00	0.00	0.00	0.00	0.00	0.00	0.00
G	0.54	0.00	0.00	0.00	0.00	0.00	0.00	0.02	0.01	0.01
	0.75	0.00	0.00	0.00	0.00	0.00	0.00	0.04	0.02	0.01

	0.25	0.00	0.00	0.00	0.00	0.00	0.00	-0.02	-0.02	-0.02
BCπ0	0.54	0.00	0.00	0.00	0.00	0.00	0.00	0.02	0.02	0.02
	0.75	0.00	0.00	0.00	0.00	0.00	0.00	0.04	0.02	0.03

BSSVS	0.25	0.00	0.00	0.00	-0.01	0.00	0.00	0.01	0.01	0.02
	0.54	0.00	0.00	0.00	0.00	0.00	0.00	0.02	0.02	0.02
	0.75	0.00	0.00	0.00	-0.01	0.00	0.00	0.04	0.03	0.03

Differences are for scenarios 1 and 2 across generations and different values of the genetic correlation (r_g_) between both traits

^1^Generations 1, 2, 3 and 4; ^2^animals with phenotypes for: both traits (1&2), only trait 1 (1) or neither of the traits (no)

**Table 4:** Increase in accuracy comparing MT to ST models for trait 2

		Scenario 1				Scenario 2				
		
		**1**^**1**^	2	3	4	1	2	2	3	4
Model	**r**_**g**_	**1&2**^**2**^	1&2	no	no	1&2	2	no	no	no
	0.25	0.00	0.01	0.00	0.00	-0.01	-0.01	0.03	0.00	0.00
A	0.54	0.02	0.02	0.01	0.01	0.01	-0.01	0.10	0.03	0.01
	0.75	0.05	0.05	0.03	0.02	0.05	0.00	0.20	0.06	0.04

	0.25	0.00	0.00	0.00	0.01	0.00	0.00	0.02	0.01	0.01
G	0.54	0.02	0.02	0.02	0.02	0.02	0.01	0.07	0.04	0.03
	0.75	0.04	0.04	0.04	0.05	0.04	0.02	0.13	0.07	0.07

	0.25	0.01	0.01	0.01	0.02	0.00	0.00	0.02	0.01	0.01
BCπ0	0.54	0.02	0.02	0.03	0.03	0.02	0.01	0.08	0.05	0.05
	0.75	0.04	0.04	0.05	0.06	0.05	0.02	0.14	0.08	0.09

BSSVS	0.25	0.01	0.01	0.02	0.03	0.00	0.00	0.03	0.03	0.04
	0.54	0.02	0.02	0.03	0.04	-0.01	-0.02	0.06	0.03	0.04
	0.75	0.04	0.04	0.05	0.06	0.04	0.02	0.14	0.09	0.10

Differences are for scenarios 1 and 2 across generations and different values of the genetic correlation (r_g_) between both traits

^1^Generations 1, 2, 3 and 4; ^2^animals with phenotypes for: both traits (1&2), only trait 2 (2) or neither of the traits (no)

**Table 5:** Significance of differences in accuracies between ST and MT models

Model	Scenario	r_g_	Trait 1	Trait 2
G	1	0.25		
G	1	0.54		**
G	1	0.75		***
BCπ0	1	0.25		
BCπ0	1	0.54		***
BCπ0	1	0.75		***
BSSVS	1	0.25		
BSSVS	1	0.54		**
BSSVS	1	0.75		***

G	2	0.25		
G	2	0.54		***
G	2	0.75	**	***
BCπ0	2	0.25		
BCπ0	2	0.54		***
BCπ0	2	0.75	*	***
BSSVS	2	0.25		
BSSVS	2	0.54		
BSSVS	2	0.75	**	***

Comparisons are between the accuracy of ST and MT implementations of the same SNP-based models for animals without any phenotypes (generation 3) across scenarios, genetic correlations (r_g_), and traits

*** P-value < 0.001, ** P-value < 0.01, * P-value < 0.05

For trait 2, the accuracy increased with the MT model in nearly all the situations (Table 4). For animals with phenotypes, a maximum increase in accuracy of 0.05 was observed for both scenarios 1 and 2. For the SNP-based models, maximum increases in scenario 2 were as high as 0.14 for animals that had phenotypes only for trait 1, and 0.09 for animals without any phenotypes. For the first generation of juvenile animals, nearly all the MT models gave significantly higher accuracies for trait 2, when the genetic correlation with trait 1 was 0.54 or higher (Table 5).

All MT models showed a higher increase in accuracy for trait 2 for animals with only phenotypes for trait 1 compared to animals without any phenotypes. For those animals with only phenotypes for trait 1, the highest increase in accuracy was 0.20 obtained with model A, compared to 0.13-0.14 with G, BCπ0 and BSSVS models. In addition to this result, Figure 4 shows that for the accuracy of trait 2, at genetic correlations of 0.25 and 0.54, having genotypes for the animals is more effective (generation 3_nophen; model G, BCπ0 and BSSVS) than having phenotypes for trait 1 (generation 2_nophen; model A). However, to achieve a high accuracy for trait 2 at a genetic correlation of 0.75 having phenotypes for trait 1 is more effective than having genotypes.

### Bias and (co)variance of estimated breeding values

Table 6 shows the coefficients of regression of the simulated on the estimated breeding values for the first generation of animals without phenotypes, across both traits and all models and for scenarios 1 and 2. The regression coefficients were all close to 1.0. This indicates that there was generally little bias in the estimated breeding values.

**Table 6:** Coefficients of regression of simulated on estimated breeding values.

				ST			MT	
			
Trait	Scenario	Model	0.25	0.54	0.75	0.25	0.54	0.75
1^1^	1	A	1.02			0.96	0.96	0.96
	1	G	1.02			0.99	0.99	0.99
	1	BCπ0	1.00			1.00	1.00	1.00
	1	BSSVS	0.99			0.97	0.99	0.99
	
	2	A	1.01			0.94	0.94	0.92
	2	G	1.00			0.98	0.98	0.99
	2	BCπ0	1.00			0.93	1.01	0.99
	2	BSSVS	1.00			0.98	1.01	0.99

2	1	A	1.01	0.97	1.00	1.00	0.96	0.97
	1	G	1.00	0.98	1.01	1.00	0.98	1.00
	1	BCπ0	1.00	0.99	1.02	1.02	1.00	1.01
	1	BSSVS	1.01	1.00	1.03	1.01	0.97	0.97
	
	2	A	1.01	0.99	0.99	1.06	1.01	0.98
	2	G	1.04	1.02	1.02	1.03	1.02	1.02
	2	BCπ0	0.97	0.98	0.97	0.98	1.07	1.03
	2	BSSVS	0.97	0.99	0.99	1.07	1.08	1.07

Regressions are performed for the ST or MT analyses, for animals without any phenotypes (generation 3), averaged across 25 replicates

^1^For trait 1 each ST model was only run once, because trait 1 was simulated independently of the genetic correlation

Table 7 shows the correlation between estimated breeding values of traits 1 and 2 for the first generation of animals without phenotypes (generation 3), across models and scenarios 1 and 2. In all situations, this correlation was lower than the genetic correlation for the ST models, and higher than the genetic correlation for the MT models. For the ST models, the correlations in scenario 1 were closer to the genetic correlations than those in scenario 2. The results from scenario 1 showed that the correlations between estimated breeding values of the two traits from the MT models were closer to the simulated genetic correlations, when SNP-based models were used, compared to the purely polygenic model A. The correlations for model A were higher than the simulated values, despite the fact that genetic correlations estimated in the model were very close to the simulated correlations (results not shown).

**Table 7:** Correlations between estimated breeding values for trait 1 and 2

			ST			MT	
		
Scenario	Model	0.25	0.54	0.75	0.25	0.54	0.75
1	A	0.20	0.43	0.60	0.33	0.64	0.86
1	G	0.20	0.44	0.61	0.31	0.62	0.84
1	BCπ0	0.20	0.44	0.60	0.31	0.63	0.84
1	BSSVS	0.20	0.43	0.59	0.32	0.63	0.83

2	A	0.15	0.32	0.44	0.41	0.73	0.91
2	G	0.19	0.37	0.53	0.36	0.65	0.87
2	BCπ0	0.19	0.37	0.51	0.35	0.68	0.86
2	BSSVS	0.19	0.36	0.51	0.37	0.70	0.90

Breeding values are obtained from ST or MT models, for animals without any phenotypes (generation 3), averaged across 25 replicates

## Discussion

The objectives of this paper were to develop methods to apply MT genomic breeding value prediction, and to evaluate their impact on the accuracies of obtained breeding values compared to ST genomic breeding value prediction. In the simulations, we assumed an effective population size of 500. This number is higher than the effective population size in current livestock populations, but was primarily chosen to obtain levels of LD, in relation to the distance between markers, that are comparable to that in livestock populations. As a result the accuracies of the ST analyses were somewhat lower than those in other simulation studies where an effective population size of 100 was assumed e.g. [5,9,13]. When MT instead of ST SNP-based models were used, in nearly all the cases, the accuracy of prediction did increase with a maximum increase for the second trait of 0.14. This is in line with a simulation study that showed that an across-country model G for dairy cattle yielded higher accuracies than a model including information from only one country [22].

### Parameterization of the model

The models applied here allowed for increasing complexity levels of the assumed underlying genetic architecture. Model A considers the infinitesimal model, where an infinite number of loci with infinite small effects are assumed. All other models consider a finite locus model, where the number of loci is the number of SNP used. Models G and BCπ0 assume that the (co)variance of all SNP is equal. Model BSSVS assumes that there is a distribution with large effects to model SNP that are associated with a QTL and a distribution with small effects to model SNP that are not associated with a QTL. In this sense, only model BSSVS incorporates a variable selection step, which can actually be used for QTL mapping purposes e.g. [11,23]. Therefore, it was expected that model BSSVS had the greatest flexibility to fit the SNP effects, followed by models BCπ0 and G. The results confirmed this expectation, since model BSSVS generally yielded the highest accuracy, followed by BCπ0 and G models.

An important conclusion is that despite the generally consistent ranking of the models, the difference in results between the different models was generally small. Comparing our results across scenarios showed that an increase in power did result in increasing differences between the models. For instance, within all the ST analyses, the only apparent difference among models was for trait 1 in scenario 1, which was the ST analysis with the highest power. In addition, when increasing the power by performing MT rather than ST analyses, again the differences between the models were more pronounced. Several alternative scenarios could be considered that would show larger differences among the models, due to increased power: 1) a more extreme distribution of QTL effects, 2) a higher SNP density resulting in higher linkage disequilibrium between SNP and QTL, or 3) a larger reference population. Since all of these alternative scenarios are expected to increase the power to detect QTL, it was expected that the BSSVS model would achieve a higher accuracy compared to the other models.

### Computational feasibility

Given the relatively small differences found between models in our study, differences in computational demands may be an important factor that determines the model of choice in practical applications. The required computation time for the bivariate G model (281 min) was 15 times longer than for the univariate models (19 min). Bivariate G models required in ASReml on average 12.5 iterations, compared to 8.5 iterations for the ST models. Initial runs with model G implemented in a Gibbs sampler, showed that for a MT analysis of scenario 2 with an unequal number of records for both traits, a large number of iterations was required before the posterior genetic variance converged. Univariate analyses with BCπ0 and BSSVS models both required 58 min. Bivariate analyses with BCπ0 and BSSVS models both required 75 min. In both cases, a total of 10,000 cycles were run, implying that the bivariate analyses for scenario 2, which were run for 30,000 cycles, required three times as much time. These computation times imply that for the Bayesian models presented it is computationally less demanding to run one bivariate analysis compared to two ST analyses. This originates from the parameterization that implies that in a MT analysis the number of effects in the scaling vector *v_jk _*is equal to the number of analyzed traits, while the number of *q_ijkl _*effects is independent of the number of traits analyzed. Importantly, the increase in calculation time when going from ST to MT models is much smaller for the Bayesian models compared to model G. This difference is expected to further increase when the number of records used in the analysis increases, because the size of the G matrix and therefore the size of the left-hand sides of the mixed model equations increases quadratic with the number of animals, while the number of calculations in the Bayesian models increases less than linearly.

In current applications of genomic selection in dairy cattle, the number of animals included in the reference population may be as high as 16,000 [24]. Inversion of the G matrix in such cases is already challenging for ST models, and solving the mixed model equations will be even more demanding for models including multiple traits. Although computation time of models using a G matrix may be heavily affected by the applied computing strategy e.g. [25], models that are parameterized based on the numbers of loci instead of the number of animals, eventually will have a lower computational burden. Based on our results, for practical applications with rapidly increasing reference populations, using models that are parameterized based on the number of markers is preferable. Moreover, running the presented Bayesian models in an MT rather than an ST form actually reduced the total required computation time. In our study, all the models estimated breeding values and variance components simultaneously. Further reductions in computation time could be achieved by performing a typical BLUP (best linear unbiased prediction) analysis with fewer iterations to estimate breeding values, using predetermined variance components. Those variance components may be re-estimated periodically using a reduced dataset to reduce computational burden.

### Impact on the design of breeding programs

When the aim is to improve accuracy of prediction for traits that are scarcely recorded, different strategies can be adopted with regard to the selection candidates: 1) using pedigree indexes for the indicator trait and/or the trait of interest, 2) recording the performance of an indicator trait in common sib or progeny testing schemes, 3) recording performances for the trait of interest, 4) obtaining genotypes, and 5) using various combinations of these strategies. An important question is which strategy is most effective, depending on the genetic correlation with the indicator traits. For instance, in our simulation, we can compare the results of scenario 2, for animals in generation 2 that have only phenotypes for trait 1 evaluated with multi-trait model A, with the results for animals with no phenotypes in generation 3 that were evaluated with the MT SNP-based models (Figure 4). In the first situation, the parents had phenotypes for both traits, and the selection candidates had phenotypes for the indicator trait. In the second situation, the parents had phenotypes for trait 1, and half of the parents had phenotypes for trait 2, while the selection candidates were genotyped. In this situation, our results show that when the genetic correlation with the indicator trait is below ~0.5, and some animals in the reference population have records for the trait of interest, having genotypes is more effective for selection candidates than having phenotypes for the indicator trait. When the genetic correlation with the indicator trait is high (> 0.5), having phenotypes for the indicator trait is more effective, but if selection candidates are genotyped as well, the accuracy is increased by ~0.03. These findings have important implications when considering the use of genotypes to predict the breeding value of an expensive or difficult to measure trait directly, using estimated SNP effects from a limited reference population, compared to the traditional alternative using easy-to-measure correlated indicator traits. For the above comparison based on our study, when the indicator trait has a genetic correlation lower than 0.5 to the trait of interest, obtaining genotypes seems to be more effective than obtaining phenotypes for an indicator trait. It should be noted that this conclusion cannot be directly generalized to for instance scenarios where measurements are done directly on the phenotypic level and the heritability of the phenotypes used is much lower than that in our study. For other scenarios, heritabilities of the evaluated traits, as well as numbers of animals in the reference population, need to be considered to establish below which level of genetic correlation, genotyping is more effective than obtaining phenotypes for an indicator trait.

### Impact on the concepts of genetic correlations

The BSSVS model allows deviating from the assumption that, in traditional MT selection models, a large number of genes, all having infinite small effects, underlie each trait. In the infinitesimal model, a genetic correlation between two traits arises due to a subset of genes that have an effect on both traits [26]. The BSSVS model allows the analysis of scenarios in which a limited number of genes with large effects may heavily influence the genetic correlation between two traits. When investigating the basis of a genetic correlation, an important question is to determine whether a correlation arises mainly from pleiotropic effects from single genes, or from closely linked genes. It has been shown that multiple QTL models, similar to the presented BSSVS model, give a sharper indication of the QTL position [11], and a simulation study showed that it is possible to distinguish the effects of two QTL that are only 15 cM apart [27]. In other studies, it has been shown that MT QTL mapping methods may distinguish between a pleiotropic QTL versus two closely linked QTL, based on simulated [28] or real data [29]. In addition, studies based on real data confirm that multi-trait QTL mapping models have an increased power to map QTL compared to single-trait models [30]. Although the optimal model for QTL mapping may differ from the optimal model for prediction of genomic breeding values [31], an increase in power to detect QTL is expected to also yield an increase in accuracy of predicted breeding values. A study that compared published genetic correlations to correlation estimates based on reported QTL effects, generally showed a poor match between both estimates [32]. Several reasons may have led to this result, such as bias in estimated QTL effects, low resolution in mapping experiments, and statistical problems by combining results from multiple models. Multi-locus models tackle the problem of multiple testing, and thereby directly control the explained genetic (co)variance by the SNP. The three SNP-based models presented in our study, are all multi-locus models. We consider that the correlation between the estimated breeding values for the MT models is a proxy for the genetic correlation used in the model. The results for scenario 1 show that for models G and BCπ0, the correlations between estimated breeding values of both traits were similar but higher than the simulated genetic correlation. The correlation for model BSSVS was, especially at higher genetic correlations, closest to the simulated genetic correlation (Table 7). This suggests that possible bias in estimated genetic correlations depends on the ability of the model to resemble the distribution of effects of the underlying loci.

## Conclusions

New models were developed and tested for genomic selection with multiple traits. The models could deal with a scenario in which not all the animals in the reference population had phenotypes for both traits. For juvenile animals without any phenotypes, an increase in accuracy up to 0.11 was observed when using MT SNP-based models compared to an ST analysis. For animals with only phenotypes on a correlated trait, the increase in accuracy was up to 0.04 and 0.18, for genetic correlations with the trait of interest of 0.25 or 0.75, respectively. Whenever the indicator trait had a genetic correlation to the trait of interest lower than 0.5, genotyping the selection candidates yielded a higher accuracy than obtaining phenotypes for the indicator trait. However, when genetic correlations were higher than 0.5, using the indicator trait was still the best alternative. For different combinations of traits, the level of genetic correlation below which genotyping selection candidates is more effective than obtaining phenotypes for an indicator trait, needs to be derived considering at least the heritabilities and the numbers of animals recorded for the traits involved.

## Competing interests

The authors declare that they have no competing interests.

## Authors' contributions

MPLC implemented the multi-trait Bayesian models in a computer program, performed the analyses and drafted the first version of the manuscript. RFV participated in discussions on the implementation of the models and critically contributed to the final version of the manuscript. Both authors read and approved the final manuscript.
